# Supplementary material for: In Vitro and In Vivo Evidence towards Fibronectin’s Protective Effects against Prion Infection
Source: Int J Mol Sci. 2023 Dec 15;24(24):17525. doi: 10.3390/ijms242417525 (PMC10743696; doi:10.3390/ijms242417525)
Supplement: Supplementary file 1 [file ijms-24-17525-s001.zip › ijms-2690280-supplementary.pdf]

Supporting information

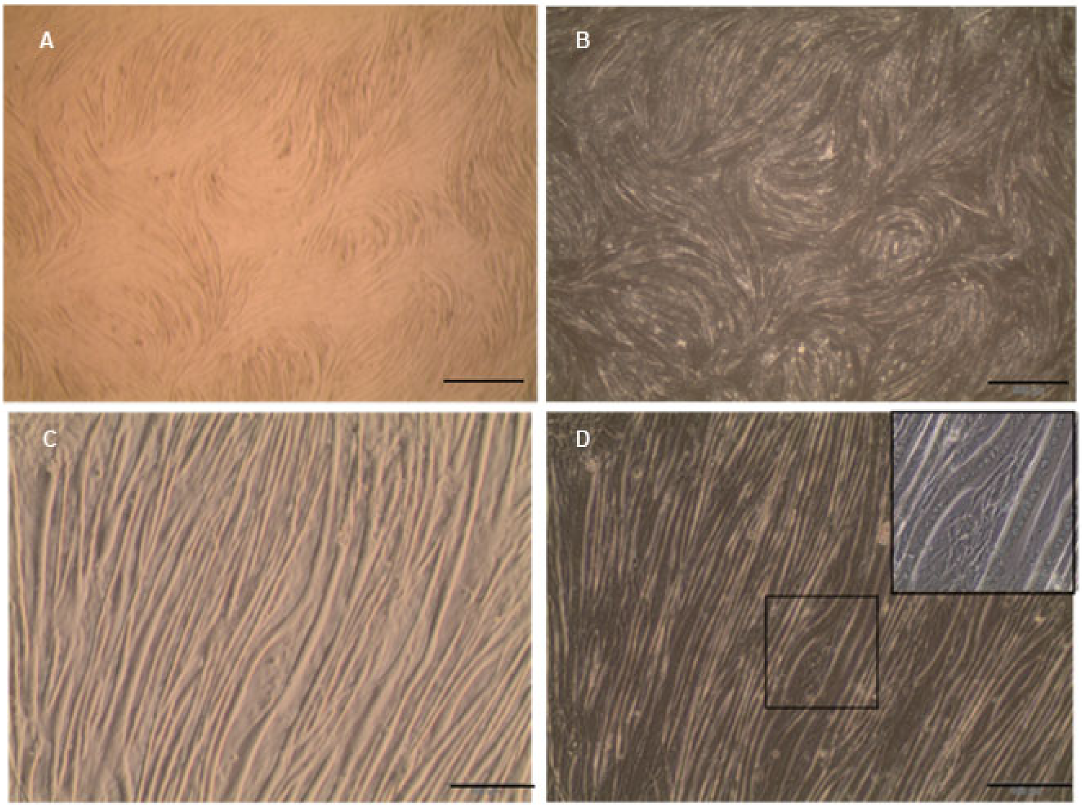

Figure S1. RML infected dC2C12 cell cultures at day 4 post inoculation observed by differential interference contrast (A, C) and phase contrast (B, D) microscopy.

A fully differentiated C2C12 culture at day 4 post inoculation reveals the long myotubes in spiral patterns (A, B). Higher magnifications of multinucleated myotubes intermingled with mononucleated cells found at the upper layer of the culture (C, D). Scale bars 500µm (A, B), 100µm (C, D).

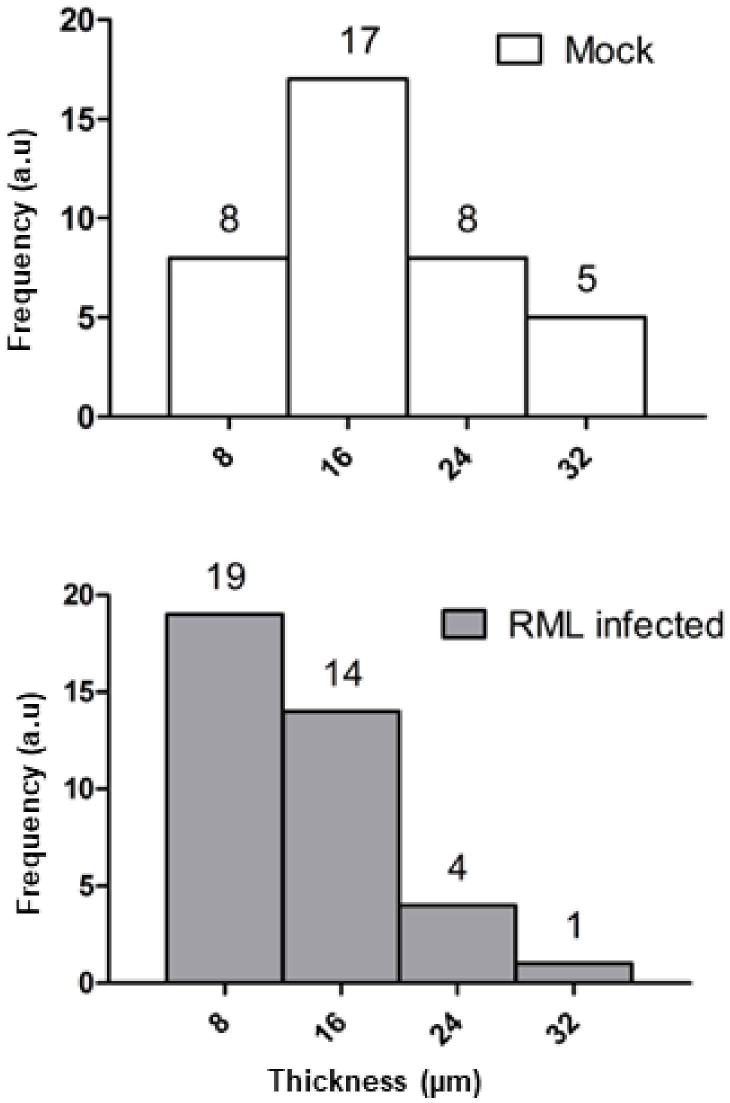

Figure S2. Histogram of myotube thickness in Mock-treated (white) and RML-infected (grey) dC2C12 culture.

Thirty-eight myotubes from a minimum of 5 micrographs, at 40x magnification, were examined per culture and each value is the average of 10 measurements per myosegment; Chi squared equals 22.021 with 4 degrees of freedom. The two-tailed P value equals 0.0002; branched myotubes were not included in the analysis.

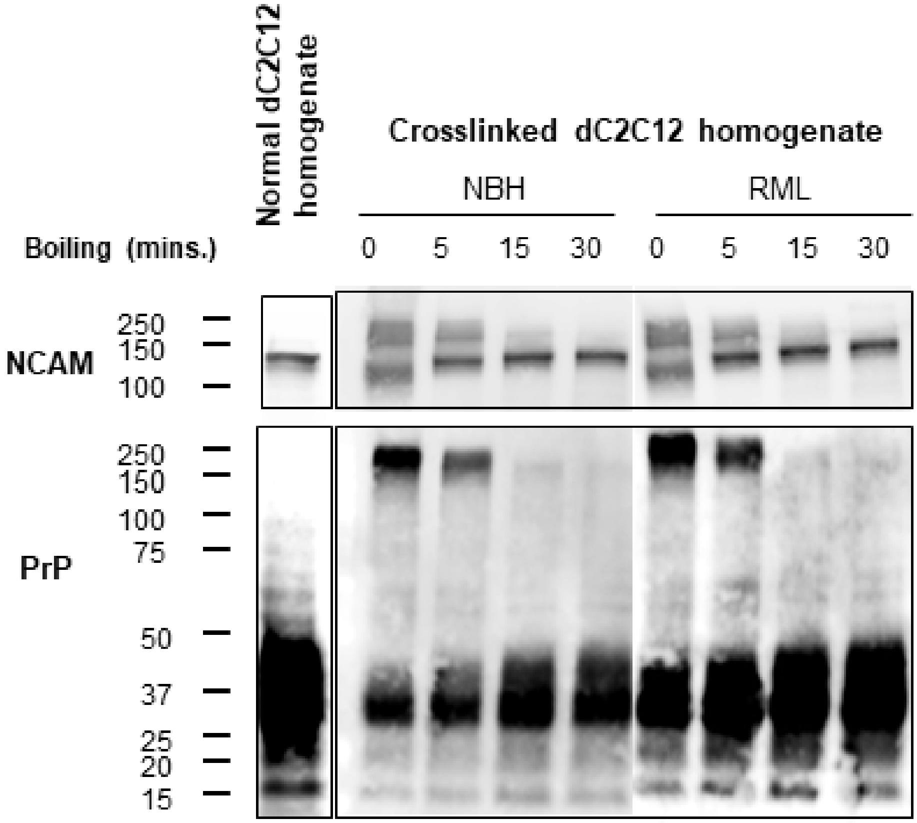

Figure S3. Immunoblot analysis of non-boiled and boiled (5, 15 and 30 min.) cell lysates after mild chemical cross-linking with 2% formaldehyde.

A complex PrP-NCAM of 200 kDa is detected with both antibodies anti-PrP and anti-NCAM that disappear in the crosslink reversal time series.

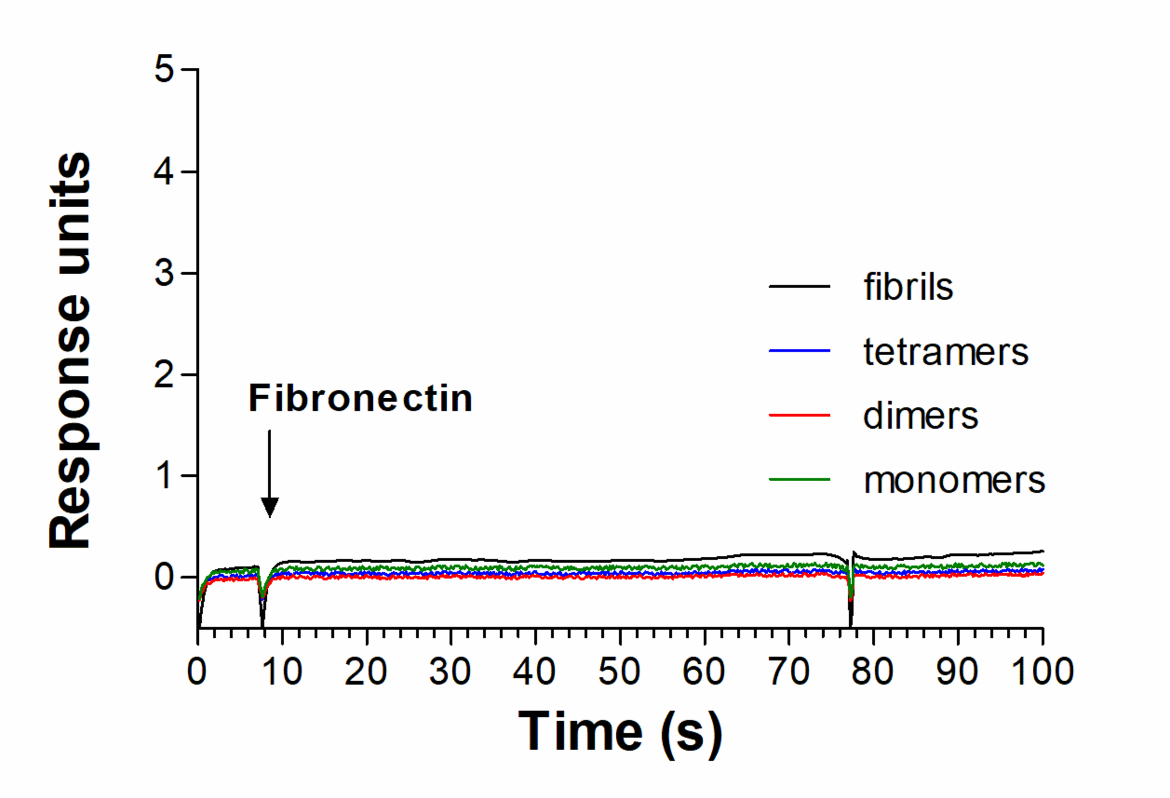

Figure S4. Interaction of recombinant PrP(23-231) with different aggregation states of fibronectin as measured by surface plasmon resonance.

Sensogram of recombinant PrP(23-231) captured on a CM5 sensor chip, interacting with monomeric, dimeric, tetrameric, and fibrillar forms of fibronectin.

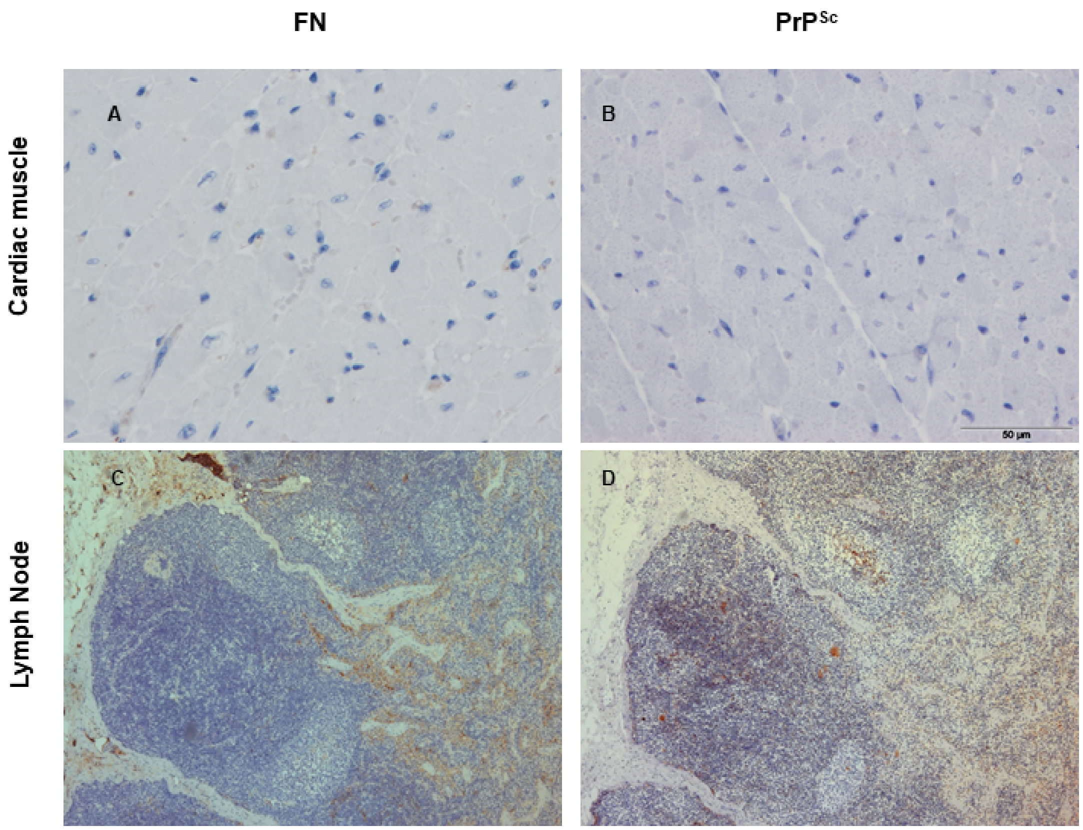

Figure S5. Immunohistochemical detection of FN and PrP^{Sc} in heart and lymph node.

No FN or PrP^{Sc} was detected in cardiac muscle (A,B). In lymph nodes (C,D), PrP^{Sc} was localized to the cortex of lymphoid follicles whereas FN was seen in the medulla associated with sinusoidal vessels. Sections A and C were incubated with anti-FN pAb (Abcam, ab2413) and sections B and D were incubated with L42 primary antibody (R-Biopharm, Darmstadt, Germany)
